# Cumulative childhood interpersonal trauma and parental stress: The role of partner support

**DOI:** 10.1177/02654075241246794

**Published:** 2024-04-15

**Authors:** Gaëlle Bakhos, Élise Villeneuve, Claude Bélanger, Alison Paradis, Audrey Brassard, Sophie Bergeron, Natacha Godbout

**Affiliations:** 1 14845 Université du Québec à Montréal, Canada; 2 7321 Université de Sherbrooke, Canada; 3 5622 Université de Montréal, Canada

**Keywords:** Childhood interpersonal trauma, dyadic analysis, parenting, parental stress, partner support, couples

## Abstract

Parents who have experienced cumulative childhood interpersonal trauma (CCIT, i.e., an accumulation of different types of abuse) tend to experience higher parental stress following the birth of a child. As CCIT is associated with lower levels of partner support, which is linked to increased parental stress, partner support could explain the link between CCIT and parental stress. Yet, these variables have never been studied using a dyadic approach. This study examined the role of received and provided partner support in the association between CCIT and parental stress. A randomly selected sample of 1119 couples with infants completed online questionnaires assessing CCIT, partner support, and parental stress. An actor-partner interdependence model path analysis showed that both parents’ CCIT were associated with increased paternal stress through fathers’ lower received and provided support, and with increased maternal stress through mothers’ received and provided support. Overall, the findings highlight the significance of examining the interdependence between both parents’ experience and the role of partner support as a key factor explaining the link between CCIT and parental stress, thereby emphasizing its importance as an intervention target.

## Introduction

Parenthood can be a rewarding, yet challenging experience. Although the arrival of a new child is typically a source of happiness, it can also be stressful for parents (Taubman Ben-Ari, 2019) as it involves multiple challenges (e.g., sleep deprivation, physiological changes, increased financial responsibilities, less personal or couple time; Nomaguchi & Milkie, 2020). Navigating the demands of parenthood can give rise to parental stress, which is recognized as the most common outcome for parents (Deater-Deckard, 2004). Parental stress could be defined as a negative response to a perceived discrepancy between parenting demands and available resources. This response arises when parents perceive their resources to be insufficient or inadequate to cope with the challenges of parenthood (Abidin, 1995). Elevated levels of parental stress are associated with adverse outcomes, such as poorer mental health (e.g., Vismara et al., 2016), strain on the couple’s relationship (e.g., Leavitt et al., 2017), and impaired child development (e.g., Neece et al., 2012). Given its detrimental effects, it would be beneficial to gain a deeper understanding of the factors linked to parental stress.

Among key factors, parents’ childhood interpersonal trauma has consistently been associated with an increase in parental stress in adulthood (e.g., Bailey et al., 2012; Hugill et al., 2017). When exposed to daily stressors, parents could experience negative emotional, cognitive, and behavioral changes that could impact couple adjustment and alter their perception of the support they give and receive in their relationships (e.g., Neff & Karney, 2009; Randall & Bodenmann, 2009). Studies examining the potential effect of partner support on the link between cumulative childhood interpersonal trauma (CCIT) and parental stress are needed, especially since current research on parental stress has typically excluded fathers and has not used a dyadic perspective (e.g., Hugill et al., 2017). In order to address these limitations, the current study used a dyadic approach to examine the indirect role of received and provided partner support in the association between CCIT and parental stress.

### Childhood interpersonal trauma

Childhood interpersonal trauma includes sexual, physical, and psychological abuse, physical and psychological neglect, witnessing interparental physical and psychological violence, and peer bullying (Godbout et al., 2009). In the past decade, research has increasingly focused on the accumulation of different types of interpersonal trauma during childhood, referred to as cumulative childhood interpersonal trauma (CCIT). Experiencing a single childhood trauma has been found to increase the risk of suffering from additional types of childhood trauma (e.g., Bigras et al., 2017; Turner et al., 2010). Childhood interpersonal trauma is recognized as an endemic problem associated with a range of negative outcomes (Dugal et al., 2016; Humphreys et al., 2020). CCIT, more specifically, has been shown to be associated with greater relational and mental health problems over time (Briere et al., 2016; Scott-Storey, 2011) than exposure to a single trauma, even when accounting for each trauma’s characteristics (e.g., severity; Bigras et al., 2017; Hodges et al., 2013).

### Cumulative childhood interpersonal trauma and parental stress

The effects of childhood interpersonal trauma may be particularly pronounced when one is adjusting to the arrival of a new child (Bailey et al., 2012; Hugill et al., 2017). Since CCIT occurs at an early stage of development, it may impede a person’s emotional, cognitive, behavioral, and relational capacities (e.g., Viezel et al., 2015), all of which are essential for successful parental adaptation. As such, increases in parental stress among CCIT survivors could be explained by the perception of lacking the necessary resources to manage the multiple demands of parenting. Previous studies have explored the cumulative nature of trauma on a range of parental outcomes (e.g., parental satisfaction, discipline, depression) and have found that CCIT accounted for greater variance in parenting difficulties than exposure to a single type of childhood trauma (e.g., Banyard et al., 2003; Cohen et al., 2008). However, empirical studies examining the link between CCIT and parental stress are scarce. The two studies having examined this issue have been conducted with a small sample of mothers facing vulnerabilities (i.e., ethnic minorities of low socioeconomic status with depressive symptoms) who had experienced interpersonal and non-interpersonal trauma, in addition to other adverse experiences in childhood and adulthood (Lange et al., 2019; Wilson et al., 2017). Additional research is needed to investigate the perspectives of both mothers and fathers, as well as the role of CCIT on parental stress in larger, more representative samples.

Most studies assessing the impact of CCIT have focused on mothers, neglecting fathers and parents’ interdependency (e.g., Bailey et al., 2012; Pereira et al., 2012). Empirical evidence shows that childhood maltreatment has implications for both survivors and their partners (Dugal et al., 2020), and that the stress encountered by one parent can affect the stress levels of the other parent (Bai & Han, 2016; Bolger et al., 1989). This stress can also be influenced by each parent’s perception of the support they give and receive in their relationship. Thus, investigating the interdependence of parents’ CCIT, partner support, and parental stress could yield more comprehensive findings.

### Partner support as a key underlying mechanism

The vulnerability-stress-adaptation model (VSA; Karney & Bradbury, 1995), originally proposed to explain variations in marital quality and stability, can provide a useful theoretical framework for conceptualizing the processes underlying the association between CCIT, partner support, and parental stress. This model puts forward three components that interact with one another. It suggests that individual vulnerabilities (e.g., childhood trauma) can undermine couples’ adaptive abilities (e.g., partner support), especially when faced with adversity (e.g., arrival of a child), which could be associated with increased parental stress. Thus, following the arrival of a new child, trauma survivors may be more likely to perceive that they do not have the sufficient personal resources to support their partners or that they are insufficiently supported by their partners. Such perceptions may be related to higher parental stress. A couple that copes with these stressors together as a team through higher perceived mutual support could potentially report lower parental stress. Therefore, it appears central to study parental dyads’ processes and explore partner support as a mechanism underlying the link between CCIT and parental stress.

Partner support specifically refers to the actions and behaviors engaged in by both partners to help one another (Brassard et al., 2011). These include emotional (e.g., expressions of empathy and love), instrumental (e.g., providing goods and services), informational (e.g., offering guidance and advice in stressful situations), and validation (e.g., reassurance that the partner’s behaviors, feelings, and responses are appropriate) dimensions (Wills & Shinar, 2000). Partner support is crucial in adult relationships, as partners are typically the primary source of comfort and reassurance in adverse situations (Fraley & Davis, 1997). As such, partner support could dampen parental stress by fostering perceptions of having sufficient resources to face parenthood challenges together. Since the first postpartum year involves various demands and psychological stressors, examining the role of partner support on parental stress is particularly relevant during this time frame. Studies have shown that partner support is a key factor in individuals’ well-being, as it decreases overall reported stress (Kapsaridi & Charvoz, 2021; River et al., 2020) and parental stress more specifically by lightening the burdens of childcare (Sampson et al., 2015). As parents often find themselves isolated and overwhelmed (Nomaguchi & Milkie, 2020; Nowland et al., 2021), partner support could be an effective way to relieve stress (e.g., Ryan et al., 2009).

Because partners’ perceptions of received (i.e., the perception of support received from one’s partner) and provided support (i.e., the perception of support provided to one’s partner) both contribute to physical and mental well-being (Berli et al., 2021; Selcuk & Ong, 2013), it is important to measure these perceptions using a dyadic approach (Liang et al., 2001). The few existing studies having examined the relationship between childhood maltreatment and partner support have found that trauma survivors are more likely to perceive lower levels of both received (e.g., Fitzgerald & Gallus, 2020) and provided partner support (e.g., Fitzgerald et al., 2020). Moreover, there is strong evidence that childhood maltreatment is an aversive relational event leading to interpersonal difficulties throughout the life course (e.g., Godbout et al., 2009), potentially hindering individuals from establishing supportive romantic relationships. Therefore, partner support might play an indirect effect in the link between CCIT and parental stress. Yet, this postulate remains to be supported by empirical data using a dyadic design accounting for the interdependence of both parents’ data.

### Gender differences

Studies examining gender differences in partner support (e.g., Crnic & Ross, 2017; Rayce et al., 2020) and parental stress (e.g., Epifanio et al., 2015; Matvienko-Sikar et al., 2018; Taubman Ben-Ari et al., 2021) yielded mixed findings. Some studies have revealed similarities in men’s and women’s perceptions of received (e.g., Verhofstadt et al., 2007) and provided support (Ko & Lewis, 2011). However, other studies have shown notable differences, with husbands indicating that they provided and received more partner support than their wives (Ko & Lewis, 2011). As for parental stress, some studies reported no gender differences (Epifanio et al., 2015) while others reported higher stress levels in mothers (Matvienko-Sikar et al., 2018) or in fathers (Taubman Ben-Ari et al., 2021). Additional research is needed to clarify the potential links between CCIT, partner support, and stress levels in both fathers and mothers.

### Objectives and hypothesis

The current study’s overall objective was to examine the role of both received and provided partner support in the link between CCIT and parental stress, in a large community-based sample of couples who recently had a child. The first objective was to test a path analysis model examining the indirect role of partner support in the relationship between CCIT and parental stress. More specifically, the aim was to examine the actor effect (i.e., the association between a parent’s CCIT and their own perceptions of partner support and parental stress) and the partner effect (i.e., the association between a parent’s CCIT and their partner’s perception of partner support and parental stress). We hypothesized that one’s CCIT would be negatively associated with one’s received and provided partner support (actor effect), which in turn would be negatively associated with one’s parental stress (actor effect). The second objective was to examine whether the effects differed between mothers and fathers. No a priori hypotheses were posited concerning partner effects and gender differences due to the limited and inconsistent findings.

## Method

### Procedure

Couples who recently had a new child were recruited through a partnership with the Quebec Parental Insurance Plan (QPIP), the universal government program that financially supports parents during their parental leave. Participants were randomly selected from the list of parents registered with QPIP across Quebec and were invited by email or telephone to participate in the study. To be eligible, (1) one of the parents had to have given birth to the child, (2) their infant had to be eight months old or less, (3) both parents had to be at least 18 years old, (4) had to be in a relationship and cohabiting, and (5) had to be fluent in French or English. After providing consent, participants were asked to complete a series of online questionnaires hosted on Qualtrics, by themselves and without consulting their partner. Each couple who participated in the study received a $40 gift card. To ensure anonymity, an alphanumeric code was randomly assigned to each couple. To be included in the analyses, both parents had to have completed the study questionnaires. The participation rate was 59 % of eligible parents. Data were collected between January 2019 and February 2023. This study was approved by the research ethics committee of the University of Quebec in Montreal.

### Participants

Of the 1142 heterosexual couples who participated in the study, 23 did not meet all the inclusion criteria. Therefore, the final sample consisted of 1119 couples aged between 19 and 57 years (*M* = 31.12, *SD* = 5.74). Table 1 presents the sample’s sociodemographic characteristics. On average, participants had two children (*M* = 1.73, *SD* = .99), and their infant was 2.64 months old (*SD* = 1.57). Most participants primarily spoke French (81.3%) and were in their current relationship for an average of 6.96 years (*SD* = 4.08; ranging from 1–21 years).

**Table 1.** Sample’s sociodemographic characteristics.

Characteristics		%	*n*
Place of birth	Canada	83.1	1859
	Europe	5.5	124
	Africa	4.6	104
	Asia	1.7	39
	South America	2.0	45
	Middle East	1.1	24
	Other	1.9	42
	Missing	.05	1
Level of education	Primary/high school	18.6	417
	Cegep (college)/professional diploma	39.2	877
	Undergraduate degree	27.5	616
	Master’s or doctoral degree	14.5	325
	Missing	.13	3
Occupation	Full-time worker	80.7	1806
	Part-time worker	7.1	158
	Student	2.7	61
	Parental leave	5.9	133
	Other (e.g., stay at home parent)	2.5	55
	Missing	.05	1
Personal annual income	$0 – $19,999	6.5	146
	$20,000 – $39,999	21.7	486
	$40,000 – $79,999	52.6	1177
	$80,000 or more	18.9	422
	Missing	.30	7
Number of children	One	50.1	1122
	Two	32.3	723
	Three	13.2	295
	Four or more	3.9	89
	Missing	.40	9
Relationship status	Common-law or cohabitation relationship	72.7	1628
	Married	27.1	606
	Missing	.10	2

*Note.* Cegep is an acronym from the French term “Collège d’enseignement général et professionnel”, which translates to General and Professional Teaching College. In Quebec, Canada, Cegep refers to a public school that provides the first level of post-secondary education.

### Measures

#### Sociodemographic characteristics and control variables

Data were collected on both parents’ age, personal income, level of education, relationship duration, infant age, and number of children. Infant health status was assessed using the question “How do you feel about your child’s health?” with response options ranging from zero – *Very fragile* to 10 – *Perfectly healthy*. The infant’s temperament was assessed using the 7-item Infant Characteristics Questionnaire (ICQ; Bates et al., 1979; Japel et al., 2000).

#### Cumulative childhood interpersonal trauma

CCIT was assessed using a French version of the Cumulative Childhood Interpersonal Trauma Questionnaire (CCTQ; Godbout et al., 2017). This 24-item measure assesses eight types of childhood interpersonal trauma experienced before the age of 18: physical, psychological, and sexual abuse, physical and psychological neglect, exposure to interparental physical and psychological violence, and peer bullying. Participants reported how often they had experienced each type of childhood trauma in each typical year before the age of 18, on a 7-point Likert-type scale ranging from zero – *Never* to 6 – *Every day or almost every day* (e.g., “One or both of my parents have slapped me in the face”; “I was intimidated or harassed by one or more children”). Childhood sexual abuse was defined as sexual contact between an adult and a child or with someone who was at least 5 years older before the age of 16, or with someone in a position of authority. Dichotomous scores were then created to indicate the presence (1) or absence (0) of the eight forms of trauma. To measure CCIT, these scores were then summed to obtain a continuous variable ranging from zero (i.e., absence of trauma) to 8 (i.e., having experienced all types of trauma). Cronbach’s alpha coefficients indicated satisfactory internal consistency in previous studies (e.g., Bigras et al., 2017; Dugal et al., 2020), as well as in the current study (α = .90, for both fathers and mothers).

#### Parental stress

Parental stress was assessed using the French version of the 18-item Parental Stress Scale (Bakhos et al., 2023; Berry & Jones, 1995), measuring the levels of stress associated with parenting. This questionnaire contains positive (e.g., “I’m happy with my role as a parent”) and negative items (e.g., “I feel overwhelmed by the responsibilities of being a parent”), with response options ranging from 1 – *Strongly disagree* to 5 – *Strongly agree*. Item scores were summed to obtain total scores ranging from 18 to 90, where higher scores indicated higher levels of parental stress. Scale validation studies (α ranging from .83 to .87, Bakhos et al., 2023; Berry & Jones, 1995) as well as the current study (α_mothers_ = .85; α_fathers_ = .88) indicated satisfactory internal consistency.

#### Partner support

The 8-item Romantic Support Questionnaire (RSQ; Brassard et al., 2011) is comprised of the Received Support and Provided Support subscales. Participants rated the degree of received and provided emotional, instrumental, informational, and validation support in their current relationship on a scale ranging from 1 – *Never* to 5 – *Always* (e.g., “My partner encourages me when I need it”; “I encourage my partner when he/she needs it”). Subscale scores were averaged, with higher scores indicating higher levels of perceived support. Internal consistency was satisfactory for men and women in the validation study (α ranging from .82 to .86; Brassard et al., 2011) as well as in the current study (received support α_mothers_ = .87; α_fathers_ = .91; provided support α_mothers_ = .85; α_fathers_ = .90).

### Data analysis

Descriptive analyses and Pearson correlations were conducted using SPSS, version 28. Paired *t*-tests and chi-square tests were performed to examine prevalence rates across genders for each type of childhood interpersonal trauma as well as CCIT, parental stress, and partner support. The percentage of missing data on these measures ranged between .3 % and 1.4 %. Data were missing at random (Little’s MCAR χ2 = 51,960, *p* = .19). Missing data were handled using the full-information maximum likelihood in Mplus (Muthén & Muthén, 2021). The absolute kurtosis and skewness were respectively below 2.3 and 7.0, suggesting that mothers’ and fathers’ total scores were normally distributed (Lei & Lomax, 2005), except for fathers’ provided support, which had a kurtosis value of 2.7. Therefore, the hypothesized model was estimated using the maximum likelihood approach with standard errors that are robust to non-normality (MLR).

Path analysis was conducted based on the Actor-Partner Interdependence Model (Kenny et al., 2006) using Mplus. This model considers the nonindependent aspect of the data by examining both actor and partner effects simultaneously. The omnibus test of distinguishability (Kenny et al., 2006) was performed to test whether dyad members differed by gender. In this test, a model without constraints is compared to a model in which the means, variances, intrapersonal and interpersonal covariances are constrained to be equal across genders. A significant chi-square index (*p* < .05) indicated that dyad members were distinguishable, χ^2^(19) = 112.389, *p* < .001. All actor and partner effects were then constrained progressively to be equal across parents to examine which associations differed according to gender. Constrained models were compared to the saturated baseline model using the −2 log likelihood difference test to compare models (Satorra & Bentler, 2010). To estimate indirect effects, the bootstrap method was used to simulate 10,000 samples and compute the 95 % confidence intervals. When confidence intervals do not include a value of zero, the indirect effect is deemed significant.

To account for potential confounding variables, nine sociodemographic variables were included as covariates in the integrative model (i.e., maternal and paternal age, maternal and paternal income, maternal and paternal level of education, number of children, relationship duration, as well as infant age, health, and temperament). Several fit indices were used to examine whether the data adequately fit the hypothesized model. A ratio of chi-square to degrees of freedom inferior to 5, a CFI value greater than .90, an RMSEA value below .60, and an SRMR value below .80 indicate good fit (Kline, 2016).

## Results

### Descriptive statistics

Prevalence rates for each type of interpersonal trauma reported by mothers, fathers, and the total sample are presented in Table 2. In the total sample, mean CCIT was 2.6. Comparison tests indicated that, compared to fathers, mothers reported higher rates of childhood sexual abuse (*χ*^2^[1] = 14.072, *p* < .001), psychological neglect (*χ*^2^[1] = 29.093, *p* < .001), psychological violence (*χ*^2^[1] = 10.81, *p* = .001), witnessing interparental psychological violence (*χ*^2^[1] = 10.77, *p* = .001), and CCIT (*t* [1098] = 4.20, *p* < .001). Fathers reported higher rates of physical violence than mothers (*χ*^2^[1] = 7.483, *p* < .01). Paired *t*-tests revealed that fathers reported higher levels of parental stress (*t* [1113] = −4.99, *p* < .001) and provided support (*t*[1101] = 3.93, *p* < .001) compared to mothers. No significant gender differences were found for received support (*t*[1102] = .25, *p* = .81). Bivariate correlations, mothers’ and fathers’ mean scores and standard deviations for all studied variables are reported in Table 3.

**Table 2.** Prevalence of childhood interpersonal trauma across gender and for the total sample.

Childhood interpersonal trauma	Mothers (*n* = 1119)		Fathers (*n* = 1119)		Total sample (*n* = 2238)		Missing	Diff.
	*n*	%	*n*	%	*n*	%	*n*	*p*
Sexual abuse	221	19.7	86	7.7	307	13.7	30	<.001
Physical violence	459	41.0	478	42.7	937	41.9	20	<.01
Psychological violence	391	34.9	313	28.0	704	31.5	21	.001
Physical neglect	138	12.3	176	15.7	314	14.0	22	.071
Psychological neglect	820	73.3	711	63.5	1531	68.4	21	<.001
Interparental physical violence	100	8.9	72	6.4	172	7.7	23	.735
Interparental psychological violence	453	40.5	377	33.7	830	37.1	21	.001
Bullying	500	44.7	476	42.5	976	43.6	23	.192
Childhood cumulative interpersonal trauma							20	<.001
No types of trauma	134	12.0	199	17.8	333	14.9		
1 type	199	17.8	215	19.2	414	18.5		
2 types	235	21.0	214	19.1	449	20.1		
3 types	180	16.1	163	14.6	343	15.3		
4 types	145	13	137	12.2	282	12.6		
5 types	106	9.5	87	7.8	193	8.6		
6 types	68	6.1	51	4.6	119	5.3		
7 types	29	2.6	28	2.5	57	2.5		
8 types	19	1.7	9	.80	28	1.3

**Table 3.** Descriptive data and correlations for cumulative childhood interpersonal trauma, received and provided partner support, and parental stress.

Variables	1	2	3	4	5	6	7	8
1. Mothers’ CCIT								
2. Fathers’ CCIT	.17***							
3. Mothers’ received support	−.19***	−.06						
4. Fathers’ received support	−.13***	−.24***	.31***					
5. Mothers’ provided support	−.16***	−.11***	.71***	.31***				
6. Fathers’ provided support	−.11***	−.22***	.31***	.75***	.27***			
7. Mothers’ PS	.25***	.17***	−.32***	−.17***	−.32***	−.14***		
8. Fathers’ PS	.12***	.27***	−.16***	−.46***	−.14***	−.46***	.30***	
*M*	2.76	2.44	4.26	4.26	4.43	4.35	32.03	33.57
*SD*	1.96	1.94	.69	.76	.54	.65	8.10	9.30

*Note.* CCIT = Cumulative childhood interpersonal trauma; PS = Parental stress.

*p < .05. **p < .01. ***p < .001.

### Integrative mediation model

First, direct links between CCIT and parental stress were examined. One’s CCIT was found to be positively associated with one’s parental stress for both mothers (*β* = .223, *p* < .001) and fathers (*β* = .254, *p* < .001). Results also showed partner effects between one’s CCIT and partner parental stress for both mothers (*β* = .13, *p* < .001) and fathers (*β* = .075, *p* < .001). The model explained 7.7% of the variance in parental stress for both parents.

Then, received and provided support were added to the model. For both parents, one’s CCIT was associated with one’s perceived lower received (*β*_*mothers,fathers*_ = −.203, *p* < .001) and provided support (*β*_*mothers*_ = −.137, *p* < .001; *β*_*fathers*_ = −.202, *p* < .001). Moreover, one’s perceived lower received (*β*_*mothers*_ = −.12, *p* < .01; *β*_*fathers*_ = −.224, *p* < .001) and provided support (*β*_*mothers*_ = −.135, *p* < .01; *β*_*fathers*_ = −.191, *p* < .01) was related to one’s higher parental stress. For partner effects, results indicated that one’s CCIT was associated with one’s partner’s lower provided support (*β*_mothers,fathers_ = −.085, *p* < .001). Additionally, mothers’ CCIT was associated with fathers’ perceived lower received support (*β* = −.101, *p* < .001).

**Figure 1.**
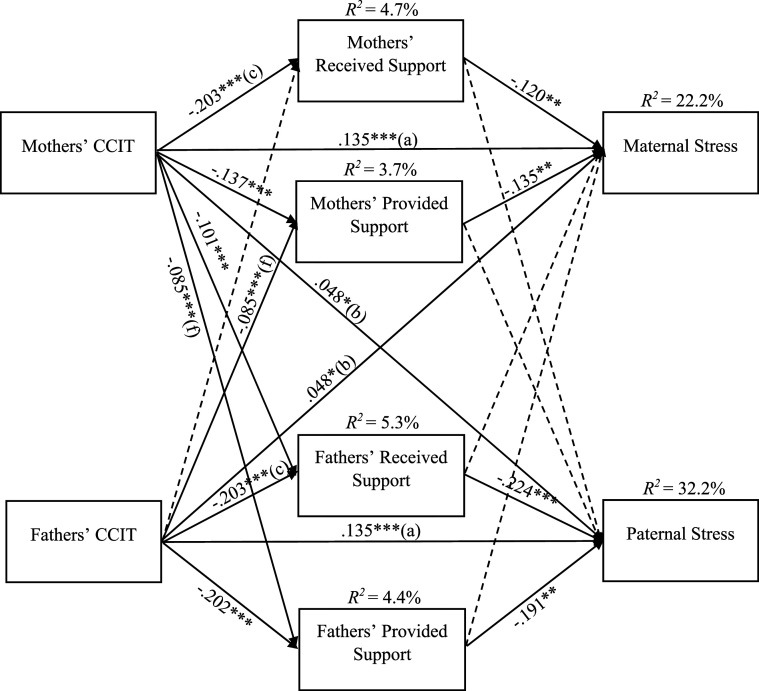
Final APIM model of the link between Cumulative Childhood Interpersonal Trauma and Parental Stress through Partner Support (Standardized Results). *Note.* CCIT = Cumulative Childhood Interpersonal Trauma; Received Support = perception of the support received from the partner; Provided Support = perception of the support provided to the partner. Identical letters (a, b, c, f) represent links that have been constrained to be equal. Covariances among the variables were estimated, but these covariances are not depicted in the figure.

Four links could be constrained to be equal for mothers and fathers (see coefficients with identical letters in Figure 1). The bootstrap method indicated seven indirect effects (see Table 4). Mothers’ CCIT was linked to maternal stress through their own lower received and provided support. Fathers’ CCIT was associated with paternal stress through their own received and provided support. Mothers’ CCIT was associated with paternal stress through fathers’ received and provided support. Fathers’ CCIT was linked to maternal stress through mothers’ lower provided support.

**Table 4.** Estimates of indirect effects, with 95% confidence intervals and significance levels.

	*Β*	Standard error	95% CI: Lower limit	95% CI: Upper limit	*p*
Actor effects					
CCIT M – RS M – Stress M	.024	.008	.008	.039	.004
CCIT M – PS M – Stress M	.019	.007	.007	.033	.009
CCIT F – RS F – Stress F	.045	.011	.027	.065	<.001
CCIT F – PS F – Stress F	.039	.011	.021	.060	<.001
Partner effects					
CCIT F – PS M – Stress M	.012	.005	.004	.022	.022
CCIT M – RS F – Stress F	.023	.007	.010	.037	.002
CCIT M – PS F – Stress F	.016	.005	.008	.027	.002

*Note.* CCIT = cumulative childhood interpersonal trauma; RS = received support; PS = provided support; Stress = parental stress; M = mothers; F = fathers; CI = confidence intervals.

Both parents’ CCIT (*β* = .161, *p* < .001) and levels of parental stress (*β* = .150, *p* < .001) were significantly correlated. Fathers’ levels of received support correlated with their own levels of provided support (*β* = .793, *p* < .001) and with mothers’ levels of provided (*β* = .161, *p* < .254) and received (*β* = .273, *p* < .001) support. Mothers’ levels of received support also correlated with their own levels of provided support (*β* = .588, *p* < .001) as well as with fathers’ levels of provided support (*β* = .290, *p* < .001). Lastly, fathers’ levels of provided support correlated with mothers’ levels of provided support (β = .230, *p* < .001).

Parents’ age, level of education, personal annual income, number of children, relationship duration, as well as infant age, health, and temperament were included as covariates. Mothers’ level of education (*β* = .111, *p* = .001) and their child’s temperament (*β* = .262, *p* < .001) were correlated to maternal stress. Fathers’ age (*β* = −.063, *p* < .05), level of education (*β* = .088, *p* = .001) as well as their child’s health (*β* = −.115, *p* = .001) and temperament (*β* = .262, *p* < .001) were correlated to paternal stress. The model was found to fit the data adequately; CFI = .905, RMSEA = .049, 95% *CI* [.043, 0.055], ratio *χ*^2^/*df =* 3.53, SRMR = .078. The final model (see Figure 1) explained 22.2 % of the variance in maternal stress and 32.2 % of the variance in paternal stress.

## Discussion

The current study tested an integrative dyadic model of the role of received and provided partner support in the link between CCIT and parental stress among parents of newborn infants. Results showed that partner support partially explained the link between CCIT and parental stress. This study extends the current knowledge on CCIT’s influence on parental outcomes in a large sample of parent dyads, while examining the interdependence of parents’ CCIT, partner support, and parental stress. Results showed the harmful and lasting direct and indirect effects of CCIT on partner support and parental stress. The findings support the use of the vulnerability-stress-adaptation model (Karney & Bradbury, 1995) in understanding parental stress, emphasizing the importance of investigating multiple dimensions of relationship function and factors that may influence it (e.g., partner support; both parents’ CCIT). These adaptive relational processes play a crucial role in coping with parental stress in the first months following the birth of a child.

The prevalence of CCIT found in the present study is congruent with that of previous studies conducted with community samples (e.g., Bigras et al., 2017). These findings confirm that childhood interpersonal trauma is an endemic problem and emphasize the importance of studying their cumulative impacts. The prevalence rates of each type of trauma are also consistent with those documented in previous studies, providing support that the current sample is representative of the general population (e.g., worldwide childhood sexual abuse rates are 18–20 % in girls and 7–8 % in boys; Stoltenborgh et al., 2015). Results indicate that most of the trauma experienced by participants occurred at the hands of their own parents, primarily in the form of psychological neglect, followed by physical and psychological violence. Nearly half of participants also reported experiencing peer bullying, highlighting the relevance of assessing trauma inflicted by both family and peers.

The integrative model supported our hypothesis that partner support acts as a key mechanism explaining CCIT survivors’ elevated parental stress. More specifically, one’s CCIT was related to one’s perception of receiving and providing less partner support, which in return was related to an increase in their own parental stress. Results are consistent with prior studies showing that trauma survivors tend to provide less support to their partners (Fitzgerald et al., 2020) and often perceive their partners as less supportive (i.e., less caring, validating, and understanding; Colman & Widom, 2004).

This finding could be attributed to the tendency of adult survivors to negatively evaluate themselves and their partners (e.g., Busby et al., 2010). This tendency might be explained by the development of negative internal working models in the aftermath of interpersonal trauma, which can shape one’s perceptions of others’ behaviors in adulthood (Baugh et al., 2019; Simpson & Rholes, 2017) and compromise the acquisition of optimal interpersonal skills (Dion et al., 2019; Godbout et al., 2019). As CCIT occurs within a context of betrayal and malevolence (e.g., from caregivers, responsible adults, or peers), it may lead to the belief that others are untrustworthy and unreliable (e.g., Baugh et al., 2019). As such, CCIT survivors may struggle to recognize the support they receive (Fitzgerald & Gallus, 2020), hindering their ability to offer support in return. They might also be inclined to choose or tolerate partners who provide low levels of support, inadvertently reproducing harmful relationship models. The demands of parenthood may also exacerbate survivors’ vulnerabilities, reducing their availability to their partners (Christie et al., 2017).

The current study also confirmed the hypothesized association between higher partner support and decreased parental stress. Such results echo those of previous studies having found that mothers’ received support is related with lower maternal stress during the first postpartum year (e.g., Negron et al., 2013; Sampson et al., 2015). As parenthood is typically navigated with one’s co-parent, who is also one’s primary source of support, feeling supported might diminish parenting strain by reducing feelings of loneliness (Lee & Goldstein, 2015) and enhancing one’s sense of control and involvement (Bouchard & Lee, 2000). The current study goes beyond the existing literature on parental stress by elucidating the role of both received and provided partner support in reducing parental stress in both mothers and fathers.

Gender differences were also found. Although higher perceived support in both parents was linked to lower parental stress, fathers reported greater levels of parental stress, and their perceived levels of received and provided support were found to have a greater influence on their own parental stress in comparison to mothers. These results can be explained by men’s greater tendency to rely on their partners for support, whereas women are more likely to report having and seeking support from a broader social network (Gurung et al., 2003). Also, studies have shown that fathers often perceive themselves as inadequate parents (Pedersen et al., 2021) and may experience delayed emotional bonding with their infants (Genesoni & Tallandini, 2009), which could increase their parental stress (Baldwin et al., 2018). Consequently, providing and receiving support may improve fathers’ sense of inclusion and involvement (Chin et al., 2011; Harmon & Perry, 2011) and reduce their paternal stress. However, the importance of mothers receiving support from their partners should not be underestimated, especially considering the societal expectations placed on mothers, as well as the specific challenges many of them face (e.g., childbirth, breastfeeding, hormonal and bodily changes). Our findings provide additional empirical support to prior research showing the importance of mutual partner support in promoting mental health.

Regarding the indirect partner effects, we found that one’s CCIT was linked to one’s partner’s lower perceived support, which, in turn, was linked to higher partner parental stress. More precisely, fathers’ CCIT was associated to mothers reporting less provided support, which in turn was related to greater maternal stress. Likewise, mothers’ CCIT was linked to fathers’ perception of providing less support, which was related to increased paternal stress.

The negative psychological outcomes of CCIT (e.g., posttraumatic symptoms, heightened emotional reactivity to stress; Seligowski et al., 2015) might intensify after the arrival of a new child. These outcomes could limit survivors’ capacity to meet their partners’ increased needs, resulting in the perception of providing less support, which could increase their own parental stress. Also, parents with CCIT may face difficulties in recognizing and expressing their own needs (MacIntosh, 2019), thereby complicating their partners’ ability to provide support. Mothers’ CCIT was also associated with fathers reporting less received support, which also increased paternal stress. Mothers with posttraumatic distress who are trying to meet their infants’ needs may have fewer resources to support their spouses. As a result, fathers may perceive less support and experience greater paternal stress. It is interesting to note that father’s CCIT was associated with mothers’ perceptions of provided support, but not with mothers’ perceptions of received support. Those results suggest that fathers with histories of CCIT may have difficulties in receiving support from their partner (e.g., due to avoidance behaviors), but may nevertheless be able to provide their partners with support. Alternately, such findings might indicate that mothers may adjust downward the support they expect from their partner with a history of trauma, resulting in lower ratings of received support.

Parents’ CCIT was directly related to their own higher parental stress levels and that of their partners, confirming previous study findings (e.g., Lange et al., 2019) and providing new insight on the effects CCIT can have on partners. Moreover, the significant correlations between mothers’ and fathers’ CCIT, as well as between mothers’ and fathers’ parental stress, suggest that CCIT survivors tend to be in relationships with other CCIT survivors and that increased stress in one parent is related to increased stress in the other. These findings provide additional evidence regarding the pairing of individuals who have experienced multiple childhood traumas, as well as the reciprocal influence of parental stress within couples (e.g., Bai & Han, 2016; Bolger et al., 1989).

Finally, the current study found a weak correlation between one’s reported received support and their partner’s perceived provided support, suggesting poor agreement between partners. This may highlight the fact that one partner may not always recognize the support provided, or that one partner may not provide support that is perceived as adequate by the other (Bolger et al., 2000; Lawrence et al., 2008). Partner differences in such perceptions have also been documented in previous studies (e.g., Vicario-Molina et al., 2015).

### Limitations and future directions

The findings should be appreciated in light of the study’s limitations. Given the study’s cross-sectional design, the analyses could not establish causation between variables. Future research would benefit from a longitudinal approach to confirm the directionality of the observed links. Further, the use of retrospective and self-reported measures may be susceptible to recall (Newbury et al., 2018) and social desirability bias (Schneider & Stone, 2015). In addition, self-reported measures may not provide a full understanding of partner support. Future research should adopt observational methods or ecological momentary assessments (EMA; daily report), and measure other forms of support (e.g., support adequacy; Lawrence et al., 2008) to better understand the links between CCIT, support, and parental stress. Furthermore, future research should examine whether these findings can be generalized to other populations facing unique challenges, including clinical populations, first-time parents, LGBTQ + parents, and single parents. Moreover, exploring additional correlates (e.g., parents’ psychological distress, other trauma such as community violence or disengaged parenting) and underlying mechanisms of parental stress (e.g., emotion regulation, romantic attachment; Bai & Han, 2016) could contribute to a better understanding of the link between CCIT and parental stress. Finally, future research could use a dimensional approach assessing the isolated effect of each type of trauma, specific combinations or sequences of trauma, or their timing, on parental outcomes.

### Implications for practice

The present study highlights the importance of promoting partner support among parents, since higher levels of parental stress can compromise parents’ mental health and relationship well-being, as well as parent-child relationships and child development (Fang et al., 2022). Partner support can be a crucial coping strategy for CCIT survivors after the birth of a new child. The development of intervention strategies that target both parents and encourage the expression of requests for support could decrease parental stress as well as discrepancies in received and provided partner support. A literature review (Monson & Fredman, 2021) showed that couple and family interventions appear promising to foster positive relationship outcomes in trauma survivors. Although existing interventions do not specifically target romantic support, their focus on key relationship mechanisms, including communication, intimacy, maladaptive belief systems, and feelings of safety (Monson & Fredman, 2021) may foster partner support and lower parental stress. Also, since partners’ perceptions of support are not highly congruent, trauma-sensitive approaches targeting mindfulness and gratitude for each parent’s contributions might improve parental well-being (Bögels & Restifo, 2014). Ultimately, the current study highlights the importance of recognizing the unique challenges experienced by mothers and fathers and suggests that partner support and teamwork are essential to foster a positive and fulfilling adjustment to parenthood.
